# Different Aberrant Changes of mGluR5 and Its Downstream Signaling Pathways in the Scrapie-Infected Cell Line and the Brains of Scrapie-Infected Experimental Rodents

**DOI:** 10.3389/fcell.2022.844378

**Published:** 2022-05-12

**Authors:** Chao Hu, Cao Chen, Ying Xia, Jia Chen, Wei Yang, Lin Wang, Dong-Dong Chen, Yue-Zhang Wu, Qin Fan, Xiao-Xi Jia, Kang Xiao, Qi Shi, Zhi-Bao Chen, Xiao-Ping Dong

**Affiliations:** ^1^ State Key Laboratory for Infectious Disease Prevention and Control, NHC Key Laboratory of Medical Virology and Viral Diseases, Collaborative Innovation Center for Diagnosis and Treatment of Infectious Diseases (Zhejiang University), National Institute for Viral Disease Control and Prevention, Chinese Center for Disease Control and Prevention, Beijing, China; ^2^ Center for Biosafety Mega-Science, Chinese Academy of Sciences, Wuhan, China; ^3^ College of Life Science and Technology, Heilongjiang Bayi Agricultural University, Daqing, China; ^4^ China Academy of Chinese Medical Sciences, Beijing, China; ^5^ College of Agricultural, Guangdong Ocean University, Zhanjiang, China; ^6^ Shanghai Institute of Infectious Disease and Biosafety, Shanghai, China

**Keywords:** mGluR5, infection, diseases, (GPCRs)-IP3-IP3R-Ca^2+^, MAPK

## Abstract

Metabotropic glutamate receptor subtype 5 (mGluR5) is a G-protein-coupled receptor found widely in the central nervous system. It has been involved in the development and progression of some neurodegenerative diseases, but its role in prion diseases is rarely described. In this study, the changes of mGluR5 and its downstream signaling pathways in prion-infected cell line SMB-S15 and the brains of scrapie-infected experimental rodents were evaluated by various methodologies. We found the levels of mGluR5 were significantly increased in a prion-infected cell line SMB-S15 and the cultured cells transiently express an abnormal form PrP (Cyto-PrP). Using immunoprecipitation tests and immunofluorescent assays (IFA), molecular interaction and morphological colocalization between PrP and mGluR5 were observed in the cultured cells. We identified that the (GPCRs)-IP3-IP3R-Ca^2+^ pathway was activated and the levels of the downstream kinases p38, ERK, and JNK were increased in SMB-S15 cells. After treated with mGluR5 antagonist (MTEP) or the removal of prion replication by resveratrol in SMB-S15 cells, the upregulations of mGluR5 and the downstream kinases were restored in a certain degree. Moreover, increased mGluR5 contributes to the cell damage in prion-infected cells. Contrarily, the levels of mGluR5 in the brains of several scrapie-infected rodent models were decreased at terminal stage. IFA of the brain sections of scrapie-infected rodents demonstrated that the signals of mGluR5 were preferentially colocalized with the NeuN-positive cells, accompanying with severe neuron losses in Nissl staining, which might be a reason for the decrease of mGluR5. Our data indicate the different aberrant alterations of mGluR5 and the downstream signaling pathways during prion infection *in vivo* and *in vitro*.

## Introduction

Prion diseases or transmissible spongiform encephalopathies (TSE) are a group of fatal neurodegenerative diseases affecting humans and various species of animals (Scheckel and Aguzzi, 2018). The causative agent is prion, a pathogenic form (PrP^Sc^) caused by abnormal conformational changes from the normal cellular prion protein (PrP^C^), which is relatively insoluble, partial protease resistant, and infectious (Sigurdson et al., 2019). The main pathological features include spongiform degeneration, reactive gliosis, neuron loss, and amyloid plaque deposition in central nervous system (CNS) (Prusiner, 1998; Wadsworth and Collinge, 2011). As a membrane-anchored protein, PrP^C^ shows active molecular interactions with many other membrane proteins, leading to activating relevant signaling pathways and inducing physiological functions of downstream factors (Aguzzi and Calella, 2009). While in the presence of PrP^Sc^, those molecular interactions may cause pathological outcomes upon the host cells.

Metabotropic glutamate receptors (mGluR) are a class of membrane proteins, belonging to G-protein-coupled receptors (GPCRs). According to the homology of amino acid (aa.) sequence, binding specificity and transduction mechanism, GPCRs consist of eight mGluRs that are divided into three groups (Conn and Pin, 1997). mGluR5, belonging to group I, is widely distributed in CNS, especially in hippocampus and cortex, mainly located in the post-synaptic membrane (Shigemoto et al., 1993; Jong et al., 2005). Plenty of studies have found that mGluR5 participates in the development and progression of neurodegenerative diseases, including Alzheimer’s disease (AD) (Abd-Elrahman and Ferguson, 2021), Parkinson’s disease (PD) (Zhang et al., 2019), and Huntington’s disease (HD) (Ribeiro et al., 2014). Recent evidences have suggested that amyloid beta oligomers (Aβo) in AD mediate synaptotoxic signaling through PrP^C^ and mGluR5, while α-synuclein-PrP^C^ interaction induces cognitive impairment through mGluR5 and NMDAR2B (Um et al., 2013; Hamilton et al., 2015; Beraldo et al., 2016; Ferreira et al., 2017). mGluR5 is known to modulate mitogen-activated protein kinases (MAPKs) signaling pathways (Mao et al., 2005; Zhang et al., 2016). mGluR5 activates phospholipase C (PLC) by coupling with Gq protein and heterotrimeric G protein. PLC hydrolyzes 4,5-bisphosphate phosphatidylinositol in cells to produce inositol 1,4,5-triphosphate (inositol 1,4,5-triphosphate, IP3) and second messenger diacylglycerol (DAG). IP3 can activate phosphoinositide receptors on the endoplasmic reticulum (ER) of cells to release the stored Ca^2+^, increase the concentration of Ca^2+^, and activate the Ca^2+^ signaling pathway, while DAG activates protein kinase C (PKC), PLA2, MAPK, and regulation of ion channels (Conn and Pin, 1997; Ireland and Abraham, 2002; Wang et al., 2020). Understanding mGluR5 signaling pathway is of great significance for understanding of its role in the pathogenesis of neurological diseases. However, the role of mGluR5 in prion disease remains unsettled.

In the present study, the potential changes of mGluR5 were evaluated in a prion-infected cell line SMB-S15 and several prion experimental animals. The levels of mGluR5 were increased in prion-infected cells and the cells transiently express abnormal Cyto-PrP. Meanwhile, the IP3-IP3R-Ca^2+^ pathway was activated and its downstream kinases p38, ERK, and JNK were increased in prion-infected cells. The molecular interaction and morphological colocalization between PrP and mGluR5 were also addressed. After treated with mGluR5 antagonist (MTEP) or removal of prion replication in cells, the upregulations of mGluR5 and the downstream kinases were restored in a certain degree. On the contrary, the levels of mGluR5 in the brains of scrapie-infected rodent models at terminal stage were decreased, especially in the regions of cortex and hippocampus. The signals of mGluR5 were preferentially colocalized with the NeuN-positive neurons in brain tissues of scrapie-infected rodent models, accompanying with severe neuron losses in Nissl staining, which may be a reason for the decrease of mGluR5.

## Materials and Methods

### Cell Culture

Cell line SMB-S15 and its control partner cell line SMB-PS were acquired from Roslin Institute, United Kingdom (Clarke and Haig, 1971). Cell line SMB-S15, a mesodermal-derived cell line, was originally established by culturing with the brain tissues taken from a mouse clinically affected with scrapie strain Chandler, in which PrP^Sc^ replication was maintained by cell passage. Cell line SMB-PS was derived from SMB-S15 cells after exposing to pentosane sulfate (PS), which was verified to be free of prion replication *in vitro* and infectivity *in vivo* (Birkett et al., 2001). Cell line SMB-RES was established from the SMB-S15 cells treated with 10 µM resveratrol for 7 days, which was verified to be free of prion replication *in vitro* and infectivity *in vivo* (Jing Wang et al., 2016). Cell lines were cultured in Dulbecco’s modified Eagle’s medium (DMEM) supplemented with 10% fetal bovine serum (FBS) at 33°C in a humidified incubator with 5% CO_2_. Human embryonic kidney (HEK) 293T cells without detectable endogenous PrP protein and cultured with DMEM were supplemented with 10% FBS in a cell incubator with 5% CO_2_ at 37°C. Mouse microglia BV2 cultured with DMEM were supplemented with 10% FBS in a cell incubator with 5% CO_2_ at 37°C.

### Drug Treatment

SMB-S15 cells were exposed to 100 µM MTEP (a selective mGluR5 antagonist, ab120035, Abcam, United States**)** for 30 min, and then stimulated by 100 μM L-glutamate (ab120049, Abcam, United States) for 1 h; SMB-S15 cells were exposed to 100 μM L-Glutamate for 1 h, and then treated by 100 µM MTEP for 1 h; SMB-PS cells were exposed to 100 μM CHPG (a selective mGluR5 agonist, ab120221, Abcam, United States) for 1 h. Various treated cells were harvested for further study.

### Cell Viability Assay

Cell viability was determined using the CCK-8 cell counting kit (CK04, Dojindo, Japan). Briefly, 4,000 cells per well were plated in a 96-well plate and incubated overnight for adherence. According to the assigned protocol, 10 μL CCK-8 reagent was added in each well at 37 °C for 4 h. Absorbance was measured at 450 nm with a spectrophotometer. Each experiment was performed in triplicate and repeated two times.

### Cell Transfection

The recombinant plasmids expressing human wild-type PrP (pcDNA3.1-PrP-PG5) and cytosolic PrP (pcDNA3.1-CytoPrP) were generated previously (Ma et al., 2002). Cells at the logarithmic growth stage were plated into six-well plates for 24 h before transfection. About 2 µg of each plasmid DNA was transiently transfected with Lipofectamine™ 3,000 (L3000150, Invitrogen, United States) according to the manufacturer’s instruction. At 48 h post-transfection, cells were harvested for further experiments.

### Preparation of Cell Lysates

Cells were harvested and centrifugated at 500×*g* for 10 min. The pellets were resuspended in a cold lysis buffer (P0013B, Beyotime, China) supplemented with the protease inhibitor cocktails set III (535140, Merck, United States), and maintained on ice for 30 min. The supernatants were collected and the protein concentrations were determined by a BCA method (71285-3, Merck, United States).

### Preparation of Brain Homogenates

Golden hamsters inoculated intracerebrally with hamster-adapted scrapie agent 263 K and C57BL/6 (C57) mice inoculated intracerebrally with mouse-adapted scrapie strains 139 A and ME7 were described previously (Gao et al., 2004; Shi et al., 2012). The average incubation times of 263 K-infected hamsters and 139A- and ME7-infected mice were 80.1 ± 5.7, 183.9 ± 23.1, and 184.2 ± 11.8 days. Five 263 K-infected hamsters and healthy controls at different time points post-inoculation were randomly selected and sacrificed; the brains were surgically removed and frozen at −80 °C for further study.

Brain homogenates were prepared based on the protocol described previously (Chen et al., 2014). Whole brain tissues were washed in TBS (10 mM Tris-HCl, 133 mM NaCl, pH 7.4) for three times, and then 10% (w/v) brain homogenates were prepared in cold lysis buffer (100 mM NaCl, 10 mM EDTA, 0.5% Nonidet P-40, 0.5% sodium deoxycholate, 10 mM Tris, pH 7.5) with protease inhibitor cocktail set III (Merck, 535,140). The tissue debris was removed with low-speed centrifugation at 2000 *g* for 10 min and the supernatants were collected for further study.

### Western Blots

Aliquots of brain homogenates and cell lysates (approx. 50 μg for each lane) were separated by 12% sodium dodecylsulfate polyacrylamide gel electrophoresis (SDS-PAGE) and electro-transferred onto nitrocellulose membranes. Membranes were blocked with 5% (w/v) skimmed milk in 1 × Tris-buffered saline containing 0.1% Tween 20 (TBST) at room temperature for 30 min and incubated with primary antibodies against mGluR5 (1:1,000, AB5675, Merck, United States), PrP (1:1,000, 6D11, Santa Cruz, United States), Gq/11α (1:1,000, 06-709, Merck, United States), IP3R (1:1,000, 8568S, CST, United States), p38 (1:1,000, 8690S, CST, United States), p-p38 (1:1,000, 4511S, CST, United States), ERK (1:1,000, 136,200, Thermo, United States), p-ERK (1:1,000, 4695S, CST, United States), JNK (1:1,000, 9252S, CST, United States), p-JNK (1:1,000, ab124956, Abcam, United States), mGluR1 (1:1,000, 12551S, CST), and β-actin (1:5,000, TA-09, ZSGB-BIO, China) at 4 °C overnight. After washing with TBST, membranes were subsequently incubated with individual HRP-conjugated secondary antibodies and reactive signals were developed using a commercial ECL kit. Images were captured by ChemiDocTM XRSC Imager (Bio-Rad, United States). To detect the presence of Protease K (PK) resistant PrP^Sc^, the cell lysates were digested with a final concentration of 20 μg/ml PK at 37 °C for 60 min prior to Western blots. The PK digestion was stopped by incubating the samples at 100 °C for 10 min.

### ELISA

The values of IP3 in the cell lysates were quantitatively measured with a commercial ELISA kit (CSB-E13410m, CUSABIO, China).

### Co-Immunoprecipitation (Co-IP)

About 1 ml of different cell lysates were mixed with 4 μg of captured antibodies and 50 μl of Dynabeads^®^-coated Protein G (10004D, Invitrogen, United States) at room temperature (RT) for 1–2 h. Subsequently, the mixtures were incubated at 4 °C overnight. The immunocomplexes were collected by separating the magnet and washed five times in washing buffer before being resolved by SDS-PAGE. The complexes were detected by further Western blots with relative detecting antibodies.

### Immunofluorescence Assay (IFA)

Brain tissues of normal and 263 K-infected hamster and 139A- and ME7-infected mice were fixed in 10% buffered formalin solution and paraffin sections (5 μm in thickness) were prepared routinely. Subsequently, brain sections were subjected to permeate with 0.3% Triton X-100 in PBS for 30 min and blocked with normal goat serum for 1 h. After blocked, sections were incubated with anti-mGluR5 (1:200, AB5675, Merck, United States), anti-mGluR1 (1:200, 12551S, CST, United States), anti-NeuN (Neuronal specific nuclear protein, 1:200, MAB377, Merck, United States), anti-GFAP (glial fibrillary acidic protein, 1:200, #3670, CST, United States), and anti-iba1 (Ion calcium-binding bridle molecule 1, 1:200, SAB2702364, Sigma, United States) in dilution solution (PBS with 2% BSA and 0.3% Triton X-100) at 4 °C overnight. The sections were subsequently incubated with 1:200-diluted Alexa Fluor 488-labeled goat-derived anti-rabbit (1:200, A11034, Invitrogen, United States) and Alexa Fluor 568-labeled goat-derived anti-mouse (1:200, A11031, Invitrogen, United States) secondary antibodies at 37 °C for 1 h. After removing secondary antibodies, DAPI were used to stain the nucleus at final concentration of 1 mg/ml at RT for 30 min (Hu et al., 2021). Slices were sealed and the images of the targeting proteins were viewed and analyzed using high-content screening system (Operetta Enspire, Perkin Elmer, United States) or confocal microscopy (LEICA TCS SP8, Germany). The integrated optical density (IOD) values of each field-specific fluorescence staining were collected automatically. The IOD values of the specific staining were determined relative to that of DAPI-specific staining.

### Immunohistochemical Staining (IHC)

Brain sections were microwaved in sodium citrate buffer at 100 °C for 30 min for antigen retrieval, and then sections were quenched for endogenous peroxidases in 3% H_2_O_2_ in methanol for 10 min before blocking with 5% BSA for 15 min at RT. The sections were incubated with anti-mGluR5 antibody (1:200, AB5675, Merck, United States) at 4 °C overnight. Subsequently, the sections were incubated with 1:250-diluted HRP-conjugated goat anti-rabbit antibody (SV0002-12, Boster, China) at 37 °C for 1 h and visualized by incubation with 3,3-diaminobenzidine tetrahydrochloride (DAB, AR1000, Boster, China). The slices were counterstained with hematoxylin (AR0005, Boster, China) for 1 min, dehydrated, and routinely mounted (Chen et al., 2020). Images were captured with an OLYMPUS BX41 microscopy with DP Controller software and quantified with Image-Pro Plus 6.0 software (Media Cybernetics).

### Quantitative Real-Time PCR (qPCR)

Real-time PCR was performed with Power SYBR Green PCR master mix (Applied Biosystems, TSE202, United States) in an ABI 7900HT Fast Sequence Detector (Applied Biosystems, United States). Total RNA was extracted from SMB cells with Trizol reagent (15596026, Gibco, United States) and then subjected to first-strand cDNA synthesis with reverse transcription system (11752050, Invitrogen, United States) according to the manufacturer’s protocol. The specific primers were designed based on the sequences of mouse mGluR5 and GAPDH genes in GenBank (NC_000073.7 and NC_000072.7). The primer sequences are as follows: GAPDH (forward: 5′-TTT​GCA​GTG​GCA​AAG​TGG​AG-3’; reverse: 5′-GAT​GGG​CTT​CCC​GTT​GAT​GA-3′) and mGluR5 (forward: 5′-CCC​TGG​TAC​CCC​TAT​CTG​CT-3’; reverse: 5′-GTC​TCT​TGG​GCA​GGT​GAT​GG-3′). PCR amplification was performed in triplicate with a total of 40 cycles (30 s at 95 °C, 30 s at 60 °C, and 60 s at 72 °C). The comparative Ct (the fractional cycle number at which the amount of amplified target reached a fixed threshold) method was used for the relative quantitative detection of the expressions of the target gene. The relative Ct for the target gene was subtracted from the Ct for the GAPDH gene using the comparative Ct method (2^-△△Ct^).

### Nissl Staining

Brain paraffin sections were stained with Nissl (1% toluidine blue) for 30 min. After rinsing quickly in distilled water, the sections were differentiated in 95% ethyl alcohol for 0.5 min (Gittins and Harrison, 2004). After dehydration, the slices were mounted with permount and observed under a microscope (Olympus BX51). The cells containing Nissl bodies were considered as neurons and counted manually.

### Statistical Analysis

Data analyses were performed using the software GraphPad Prism 7.0. Quantitative analysis of Western blots and quantification of colocalization were processed with ImageJ software. The final results were presented as mean + stand error of mean (SEM). The student’s *t* test was evaluated for statistical analysis ∗: *p* < 0.05; ∗∗: *p* < 0.01; ∗∗∗: *p* < 0.001.

## Results

### The Levels of mGluR5 Were Increased in a Prion-Infected Cell Line and the Cells Transiently Expressing Cyto-PrP

**FIGURE 1:**
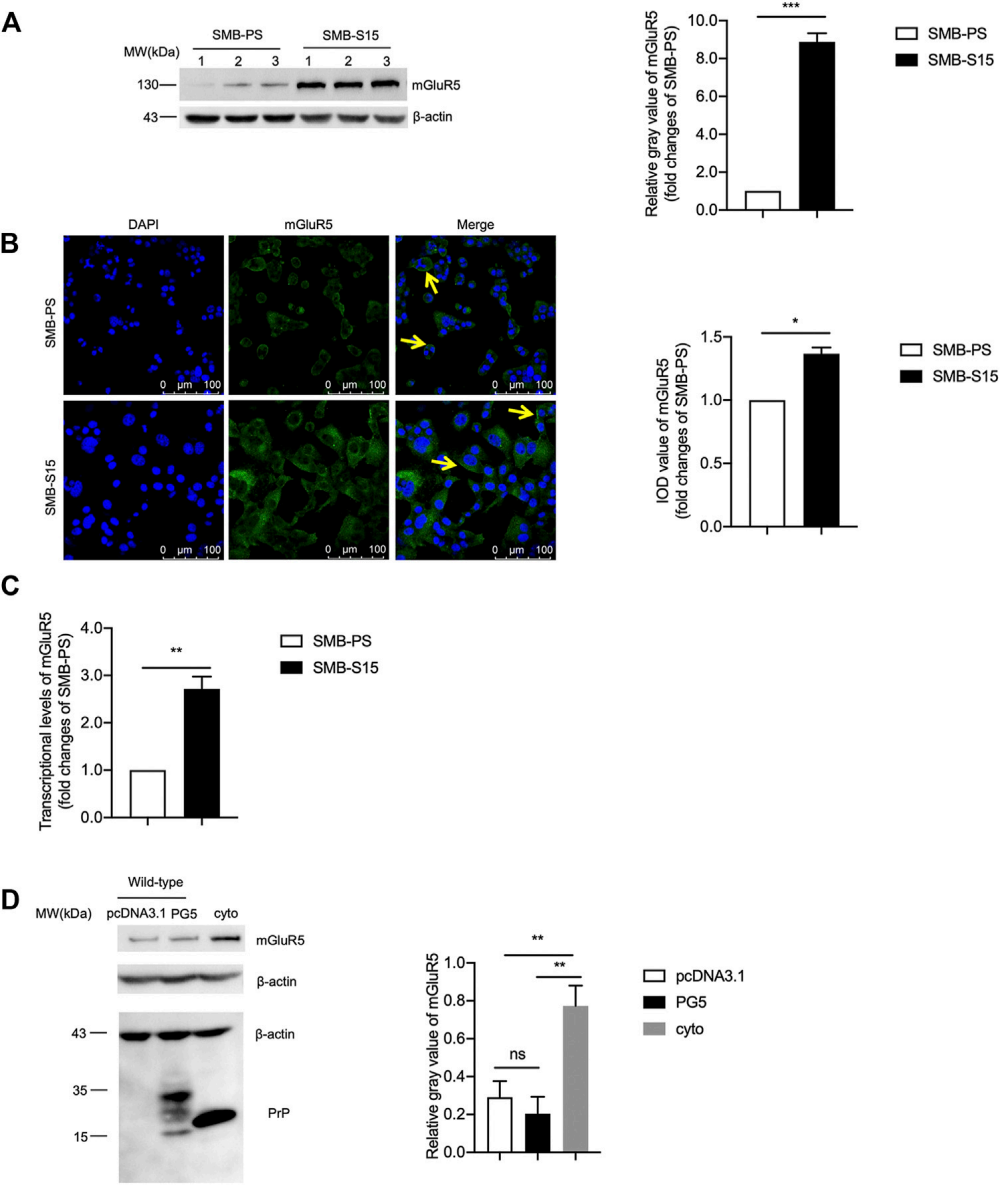
The levels of mGluR5 in a prion-infected cell line and the cells transiently expressing Cyto-PrP. **(A)** *Left:* Western blot for the levels of mGluR5 in two SMB cell lines. Relative molecular weights are marked on the left. (1, 2, 3) indicate the number of replicates. *Right:* Densitometric analyses of the average gray values of the signals of the mGluR5 after being equilibrated with that of β-actin. The relative gray value of mGluR5 in SMB-PS cells is set to 1 and *Y*-axis represents the fold changes in SMB-S15 versus SMB-PS. **(B)** *Left:* Immunocytochemical assays of the distributions of mGluR5 in two SMB cell lines. The images of DAPI (blue), mGluR5 (green), and merge (yellow arrows) are indicated above. Two SMB cell lines are indicated on the left. *Right:* The IOD analysis for the signals of mGluR5 in the images of two SMB cell lines. The quantitative results are presented as mean + SEM. The IOD value of mGluR5 in SMB-PS cells is set to 1 and *Y*-axis represents the fold changes in SMB-S15 versus SMB-PS. **(C)** Comparative analysis of the levels of mGluR5-specific mRNAs in SMB-PS and -S15 cells by real-time PCR. The transcriptional level of mGluR5-specific mRNA was determined relative to that of the individual GAPDH. The relative intensity of the transcriptions of mGluR5 gene in SMB-S15 cells is relative to that of SMB-PS cells that is set to 1. **(D)** *Left:* Western blot for the levels of mGluR5 in the cells transiently expressing wild-type PrP and Cyto-PrP. Relative molecular weights are marked on the left. *Right:* Densitometric analyses of the average gray values of the signals of the mGluR5 after being equilibrated with that of β-actin. Data are representative of three independent experiments.

To determine the possible alteration of mGluR5 along with prion accumulation, the cellular lysates of the prion-infected cell line SMB-S15 and its normal partner cell line SMB-PS were subjected into mGluR5-specific Western blots. Compared with that of SMB-PS cells, the signal of mGluR5 in SMB-S15 cells was markedly stronger, revealing remarkably increased in the relative gray values after quantitative assays with the individual data of β-actin (*p* < 0.001, Figure 1A). IFA with mGluR5 antibody illustrated more brilliant green signals in SMB-S15 cells, showing notably higher IOD value than that in SMB-PS cells (*p* < 0.05, Figure 1B). Further, total RNAs of SMB cells were prepared and the transcriptional levels of mGluR5 were comparatively assessed by real-time PCR with mGluR5-specific primers. It revealed that the average level of mGluR5 mRNA in SMB-S15 cells was about 2.5 folds of that in SMB-PS cells, showing significant difference (*p* < 0.01, Figure 1C).

To further assess the relationship between increased mGluR5 and accumulated PrP, the recombinant plasmids expressing wild-type human PrP (PG5-PrP) and Cyto-PrP were separately transfected into 293T cells that were previously verified not to contain detectable endogenous PrP (Wang et al., 2011). At 48 h post-transfection, the cells were harvested and the levels of mGluR5 were evaluated with Western blots. Compared with the control transfected with the blank vector pcDNA3.1, the mGluR5 level in the cells expressing PG5-PrP was almost unchanged while that in the cells expressing Cyto-PrP was significantly increased (Figure 1D). It seems that accumulation of either prion agent or abnormal PrP construct causes higher expression of mGluR5 in cultured cells.

Further, as the other subtype of mGluR group I, the potential changes of the mGluR1 levels in SMB cell line were assessed by mGluR1-specific Western blots. The signal intensity of mGluR1 in SMB-S15 was extremely declined compared with that in SMB-PS, highlighting remarkably decreased in the relative gray values after quantitative assays with the individual data of β-actin (*p* < 0.001, S-Figure 1A). Furthermore, SMB-PS cells were exposed to CHPG, a mGluR5 agonist, in order to simulate the statues of increased mGluR5 in SMB-S15 cells, and the signal of mGluR1 in CHPG-treated SMB-PS cells was decreased, even without statistical difference compared with that of untreated ones in the relative gray values after quantitative assays with the individual data of β-actin (S-Figure 1B). These data imply that increased mGluR5 has ability to reduce the expression of mGluR1 *in vitro*.

### Molecular Interaction and Morphological Colocalization Between PrP and mGluR5

**FIGURE 2:**
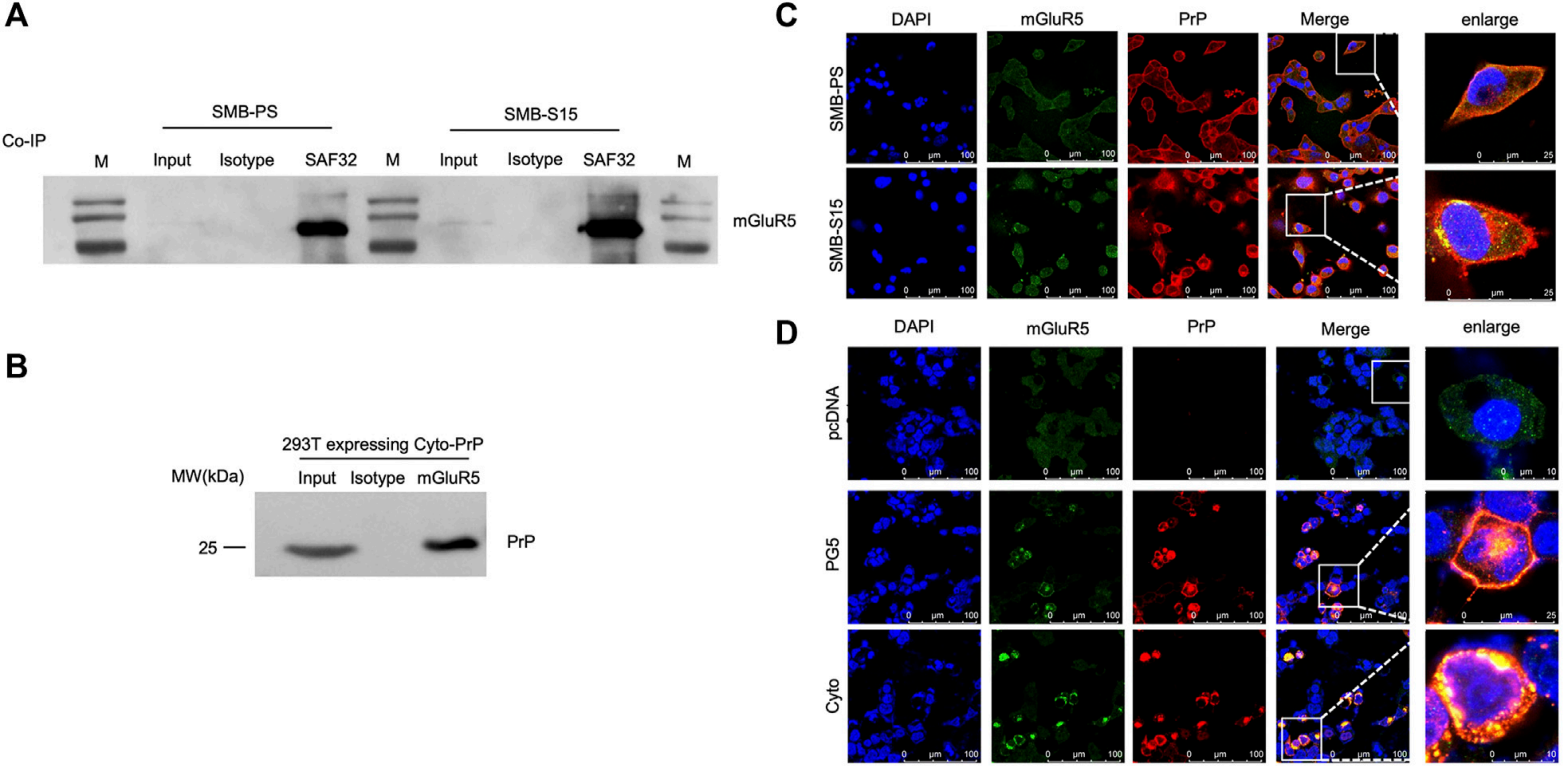
Molecular interaction and morphological colocalization between PrP and mGluR5. **(A)** Immunoprecipitation assays for the complexes of PrP and mGluR5 in the SMB-PS and -S15 cell. The cell lysates were precipitated with antibodies against PrP (SAF32), mouse IgG and were subsequently evaluated by mGluR5-specific Western blots. An aliquot of cell lysates (marked as input) was directly loaded into SDS-PAGE as controls. Two SMB cell lines are indicated above. **(B)** Immunoprecipitation assays for the complexes of PrP and mGluR5 in the 293T cell transiently expressing Cyto-PrP. The cell lysates were precipitated with antibodies against mGluR5 and mouse IgG were subsequently evaluated by PrP-specific Western blots. An aliquot of cell lysates (marked as input) was directly loaded into SDS-PAGE as controls. **(C)** Immunofluorescent assays of the co-localization between PrP and mGluR5 in SMB cells with confocal microscopy. Two SMB cell lines are marked on the left. **(D)** Immunofluorescent assays of the co-localization between PrP and mGluR5 in 293T cell transiently expressing wild-type human PrP (PG5-PrP) and Cyto-PrP with confocal microscopy. Various transfected 293T cells are marked on the left. The images of DAPI (blue), mGluR5 (green), PrP (red), and merge are indicated above. The magnification views are shown on the right each picture.

To explore the potential molecular interaction between PrP and mGluR5, the lysates of two SMB cells were employed to co-IP assay using PrP antibody (SAF32) as the capturing antibody and mGluR5 antibody as the detecting one. Clear mGluR5 bands were detected in the eluting products from both SMB-S15 and -PS cells, whereas no signal in the reactions with isotypic IgG (Figure 2A). To obtain more evidence, the lysates of 293T cells transiently expressing Cyto-PrP immunoprecipitated with mGluR5 antibody and blotted with PrP antibody. Specific Cyto-PrP signal was identified in the final eluting product of mGluR5 antibody, but not in that of isotypic IgG (Figure 2B).

To address the possible morphological colocalization of PrP and mGluR5 in cell level, SMB cells were fluorescently double-stained with PrP and mGluR5 antibodies. Confocal microscopy revealed clear colocalized signals (yellow) in the positions of cytoplasm and membrane after merged, particularly in SMB-S15 cells (Figure 2C). 293T cells transiently expressing PG5-PrP and Cyto-PrP were also subjected into double-stained IFA with PrP and mGluR5 antibodies. Amounts of colocalized signals (yellow) were identified in the cells expressing PG5-and Cyto-PrP contrast to the mock cells receiving the blank vector (Figure 2D). More colocalized signals were observed in the cells transfected with Cyto-PrP than that with PG5-PrP, especially in the cytoplasm. Those data supply the evidence of molecular interaction and morphological colocalization between PrP and mGluR5 in the cultured cells.

### Activation of G-Protein-Coupled Receptors (GPCRs)-IP3-IP3R-Ca^2+^ Pathways and Downstream Kinases in Prion-Infected Cells

**FIGURE 3:**
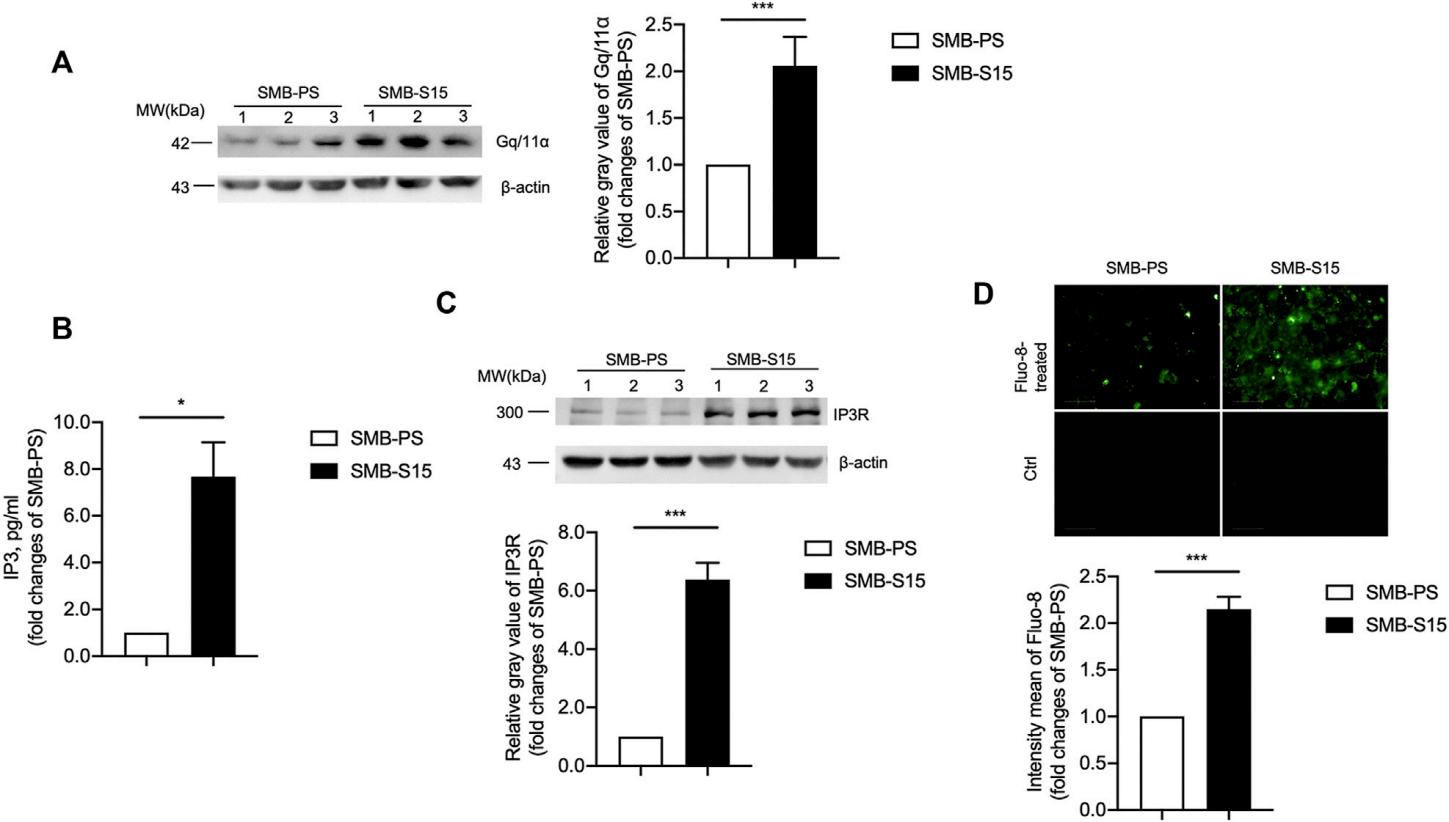
Activation of the GPCRs-IP3-IP3R-Ca^2+^ pathway in two SMB cell lines. **(A)** *Left:* Western blots for the levels of Gq/11α. Relative molecular weights are marked on the left. Two SMB cell lines are indicated above. (1, 2, 3) indicate the number of replicates. *Right:* Densitometric analyses of the average gray values of the signals of the Gq/11α after being equilibrated with that of β-actin. **(B)** ELISA for the levels of IP3. **(C)** *Top:* Western blots for the levels of IP3R. Relative molecular weights are marked on the left. Two SMB cell lines are indicated above. *Bottom:* Densitometric analyses of the average gray values of the signals of the IP3R after being equilibrated with that of β-actin. **(D)** *Top:* Immunocytochemical assays for the levels of Ca^2+^ concentration. *Bottom:* The IOD analysis for the signals of Ca^2+^ in the images of two SMB cell lines. The quantitative results are presented as mean + SEM.

The potential influence of the increased mGluR5 on the downstream GPCRs-IP3-IP3R-Ca^2+^ pathway was analyzed in two SMB cell lines. Western blot for Gq/11α, one of the subunits of GPCRs, showed significantly stronger signals in SMB-S15 than that in SMB-PS (Figure 3A). Measurement of IP3 with a commercial kit identified approximately 8-fold increase of IP3 in SMB-S15 cells compared to SMB-PS cells (Figure 3B). Western blot for IP3R revealed remarkably higher level in SMB-S15 cells (Figure 3C). Further staining of Fluo-8 on those two cell lines illustrated obviously higher fluorescent intensity in SMB-S15 cells, highlighting a higher Ca^2+^ concentration (Figure 3D).

**FIGURE 4:**
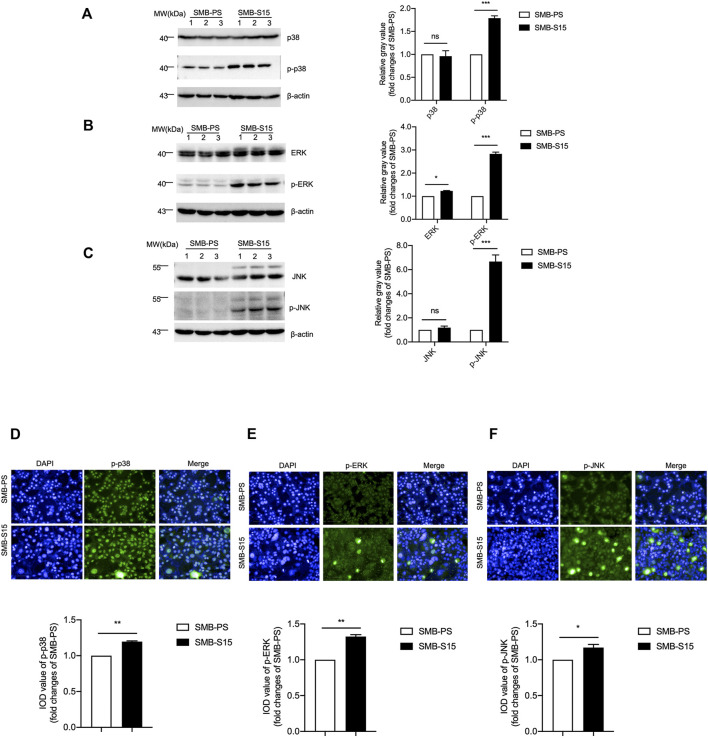
Activation of the MAPK pathway in two SMB cell lines. **(A-C)** *Left:* Western blots for the levels of p38 and p-p38 **(A)**, ERK and p-ERK **(B)**, JNK and p-JNK **(C)**. Relative molecular weights are marked on the left. Two SMB cell lines are indicated above. (1, 2, 3) indicate the number of replicates. *Right:* Densitometric analyses of the average gray values of the signals of the target proteins after being equilibrated with that of β-actin. **(D-F)** *Top:* Immunocytochemical assays of the levels and distributions of p-p38 **(D)**, p-ERK **(E)**, p-JNK **(F)** in two SMB cell lines. The images of DAPI (blue), p-38/p-ERK/p-JNK (green), and merge are indicated above. *Bottom:* The IOD analysis for the signals of p-38/p-ERK/p-JNK in the images of two SMB cell lines. The quantitative results are presented as mean + SEM. The IOD values of p-p38, p-ERK, and p-JNK in SMB-PS cells are set to 1 and *Y*-axis represents the fold changes in SMB-S15 versus SMB-PS.

The levels of several downstream kinases and their phosphorylated forms in two SMB cells were measured with Western blots with various antibodies, including anti-p38, anti-ERK, and anti-JNK. As shown in Figure 4, the levels of those three kinases were comparable between two cell lines, whereas those of the phosphorylated isoform of p38 (Figure 4A), ERK (Figure 4B), and JNK (Figure 4C) in SMB-S15 cells were higher than that in SMB-PS cells with statistical differences. Furthermore, two SMB cell lines were immunofluorescent stained with p-p38-, p-ERK- and p-JNK-specific antibodies separately, showing significantly stronger fluorescent intensities in SMB-15 cells compared to SMB-PS cells (Figures 4D–F).

**FIGURE 5:**
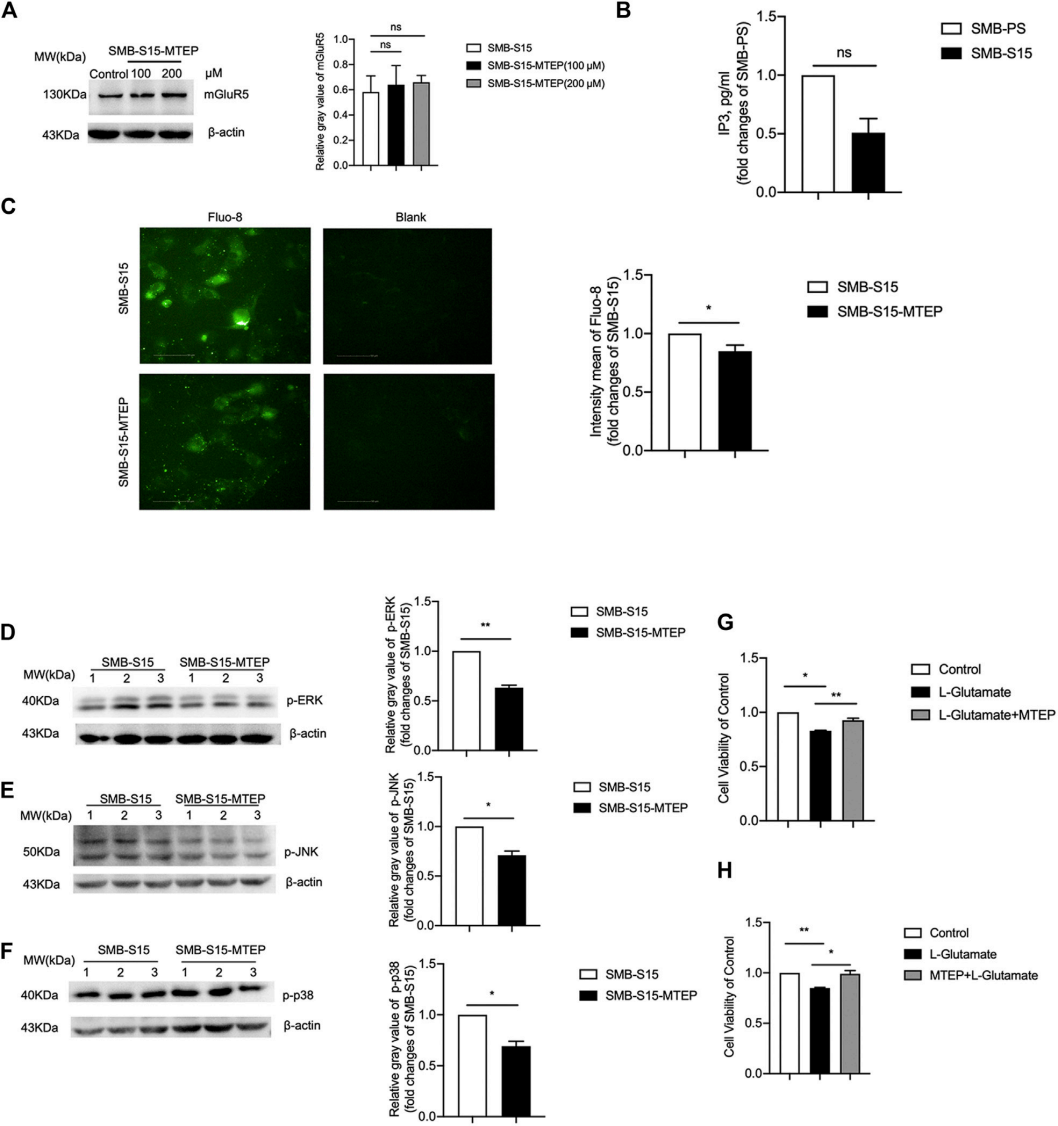
Inhibition of GPCRs-IP3-IP3R-Ca^2+^ and MAPK pathways in MTEP treated SMB-S15 cells. **(A)** *Left* ***:*** Western blot for level of mGluR5 in 100 and 200 μM of MTEP treated SMB-S15 cells. *Right:* Densitometric analyses of the average gray values of the signals of the mGluR5 after being equilibrated with that of β-actin. **(B)** ELISA for the levels of IP3. **(C)** Immunocytochemical assays for the levels of Ca^2+^ concentration. **(D-F)** *Left:* Western blots for the levels of p-ERK **(D)**, p-JNK **(E),** and p-p38 **(F)** after MTEP treatment. Relative molecular weights are marked on the left. SMB cell lines are indicated above. (1, 2, 3) indicate the number of replicates. *Right:* Densitometric analyses of the average gray values of the signals of the target proteins after being equilibrated with that of β-actin. The quantitative results are presented as mean + SEM. The IOD values of p-p38, p-ERK, and p-JNK in SMB-S15 cells is set to 1 and *Y*-axis represents the fold changes in MTEP-treated SMB-S15 versus SMB-S15. **(G,H)** Cell viability of SMB-S15 cell treated with L-glutamate only or L-glutamate and MTEP alternatively.

To verify the activation of mGluR5 on the GPCRs-IP3-IP3R-Ca^2+^ pathways after prion infection, SMB-S15 cells were treated with 100 μM or 200 μM of MTEP, a selective mGluR5 antagonist, which did not change the level of mGluR5 in Western blots (Figure 5A). Subsequently, SMB-S15 cells were exposed to 100 μM MTEP and the levels of GPCRs-IP3-IP3R-Ca^2+^ pathways were comparatively analyzed. IP3-specific ELISA assay revealed approximately 2-fold decrease of IP3 in MTEP-treated SMB-S15 cells compared with untreated ones (Figure 5B). Fluo-8 staining illustrated remarkably lower fluorescent signal in MTEP-treated SMB-S15 cells, revealing an inhibition of Ca^2+^ concentration in a certain degree (Figure 5C). Furthermore, Western blots revealed the levels of phosphorylated p38, ERK, and JNK were decreased in MTEP-treated SMB-S15 cells (Figures 5D–F).

To assess the potential influence of blocking mGluR5 in the cell survival during prion infection, SMB-S15 cells were treated with MTEP before or after exposure to L-glutamate. The cell survival was evaluated with CCK-8 assays. When compared with the mock cells without any treatment, the relative cell survival rates of SMB-S15 were lower after exposure to L-glutamate, which revealed the worsen of cell viability in the preparation of 1 h incubation, while that of SMB-S15 alternatively treated by METP before or after exposure to L-glutamate exhibited the opposite (Figures 5G,H). These data indicate that increased mGluR5 has ability to activate the (GPCRs)-IP3-IP3R-Ca^2+^ pathways and downstream kinases, and cause cell damage in prion-infected cells.

### Down-Regulation of the Levels of mGluR5 and Its Downstream Elements After Removal of Prion Replication in SMB-S15 Cells

**FIGURE 6:**
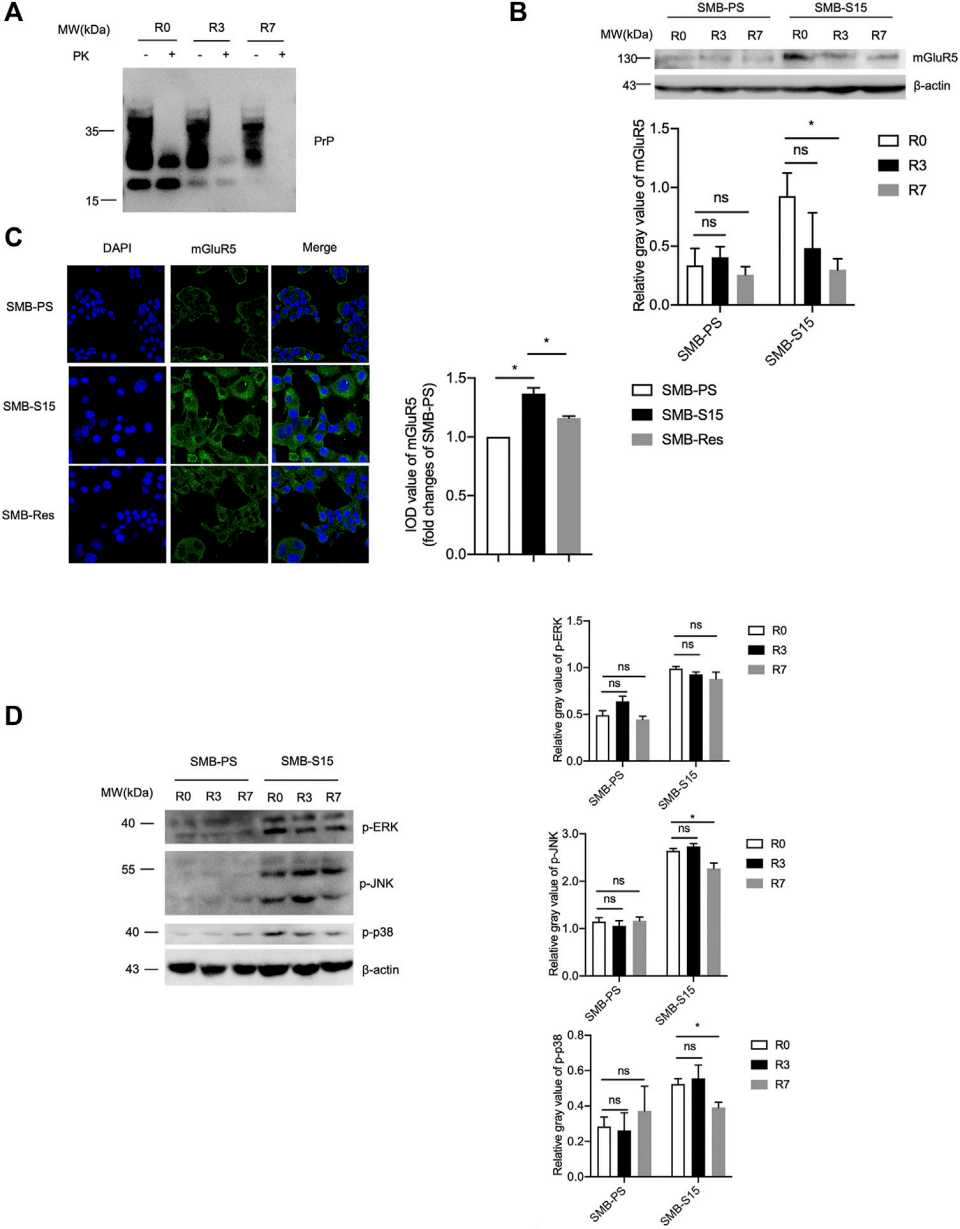
The levels of mGluR5 and its downstream elements after removal of prion replication in SMB-S15 cells. **(A)** PrP-specific Western blots of resveratrol-treated SMB-S15 cells. *R0:* The lysates of SMB-S15 cells without treatment of resveratrol. *R3:* The lysates of SMB-S15 cells receiving 10 µM resveratrol for 3 days. *R7:* The lysates of SMB-S15 cells receiving 10 µM resveratrol for 7 days. *PK* proteinase K. **(B)** *Top:* Western blots for the levels of mGluR5 in two SMB cell lines before and after removal of PrP^Sc^. *Bottom:* Densitometric analyses of the average gray values of the signals of mGluR5 after being equilibrated with that of β-actin. **(C)** *Left:* Immunocytochemical assays of the distributions of mGluR5 in various SMB cell lines. The images of DAPI (blue), mGluR5 (green), and merge are indicated above. *Right:* The IOD analysis for the signals of mGluR5 in the images of various SMB cell lines. The IOD value of mGluR5 in SMB-PS cells is set to 1 and *Y*-axis represents the fold changes in SMB-S15 versus SMB-PS. **(D)** *Left:* Western blots for the levels of p-ERK/p-JNK/p-p38 in two SMB cell lines before and after removal of PrP^Sc^. *Right:* Densitometric analyses of the average gray values of the signals of p-ERK/p-JNK/p-p38 after being equilibrated with that of β-actin.

Our previous study has verified that the treatment of resveratrol completely removes the prion replication in SMB-S15 cells (Jing Wang et al., 2016). To test the effect of removal of prion propagation on the cellular mGluR5 and its downstream factors, SMB-S15 cells were exposed to 10 µM resveratrol. Western blots showed a marked reduction of PrP^Sc^ in the cellular lysate treated for 3 days and almost undetectable PrP^Sc^ in the cells treated for 7 days (Figure 6A). Accompanying with the reduction and removal of PrP^Sc^, the level of mGluR5 in SMB-S15 cells treated with resveratrol for 3 and 7 days was also significantly reduced to the level of the untreated SMB-PS cells in Western blot (Figure 6B). mGluR5-specific IFA also identified the decreased fluorescent intensity in SMB-S15 cells treated for 7 days (Figure 6C). It indicates a synchronous reduction of PrP^Sc^ and mGluR5 in SMB-S15 cells after treatment of resveratrol.

Subsequently, the levels of the phosphorylated forms of the downstream kinases in two SMB cell lines exposed to resveratrol for 3 and 7 days were measured by the individual Western blots separately. As shown in Figure 6D, the levels of p-p38, p-ERK, and p-JNK maintained also unchanged in SMB-PS cells after exposure to resveratrol for 3 and 7 days compared to the untreated one, remaining at obviously low level. However, the levels of those three phosphorylated kinases in the resveratrol-treated SMB-S15 cells were downregulated in certain degree, particularly p-p38 and p-JNK in the cells treated for 7 days (R7), highlighting an association between cellular PrP^Sc^ and those phosphorylated kinases.

### Decreased Levels of mGluR5 in the Brains of Scrapie-Infected Rodent Models

**FIGURE 7:**
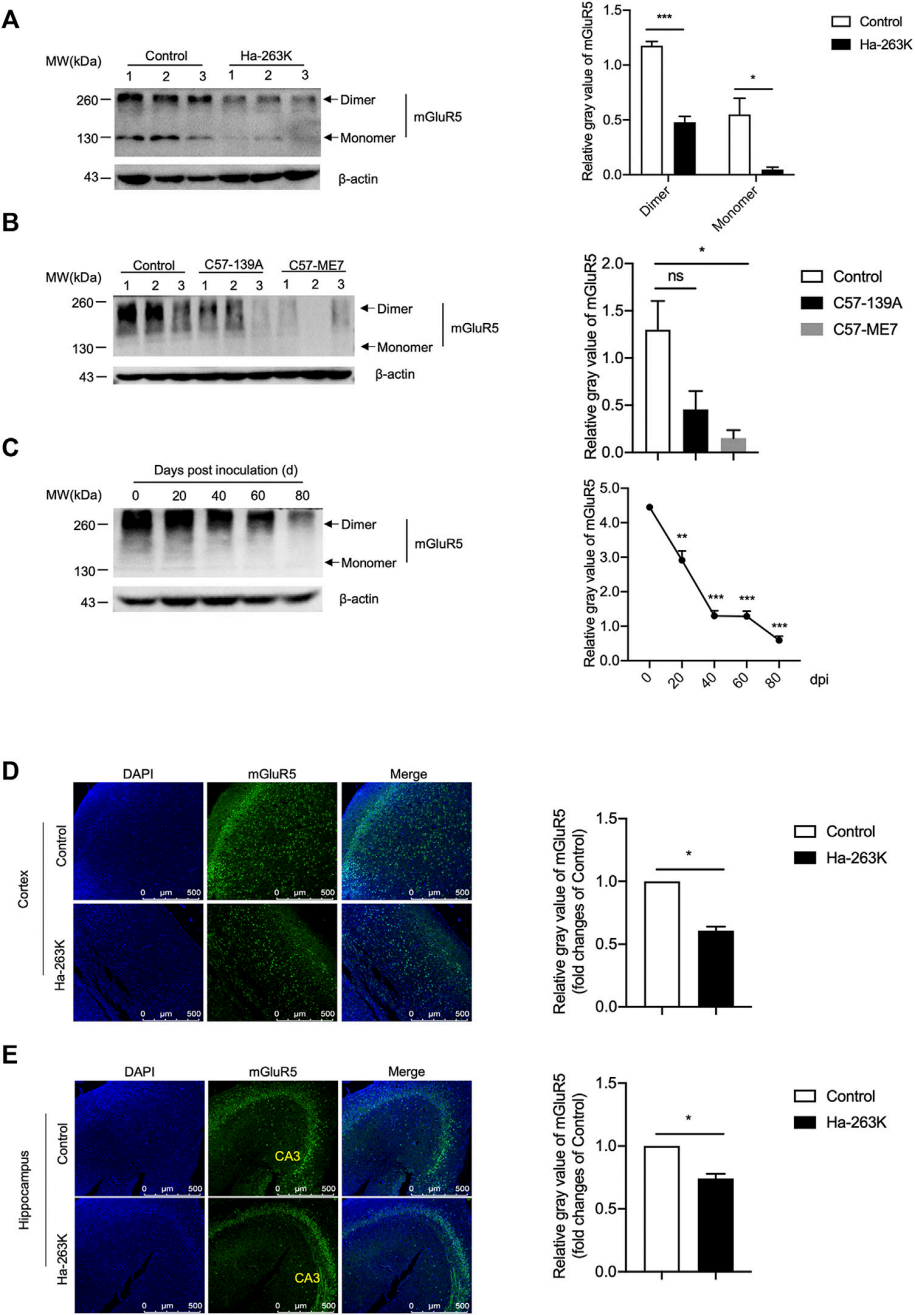
The levels of mGluR5 in the brains of scrapie-infected rodent models. **(A-B)** *Left:* Western blots for the levels of mGluR5 in brains of 263 K-infected hamsters **(A)** and 139 A or ME7-infected mice **(B)**. Various prion-infected rodent models are indicated above. **(C)** *Left:* Dynamic assays of the levels of mGluR5 in brains tissues of 263 K-infected hamsters during the incubation period. The data of dpi are shown at the top. Relative molecular weights are marked on the left. (1, 2, 3) indicate the number of replicates. *Right:* Densitometric analyses of the average gray values of the signals of the mGluR5 after being equilibrated with that of β-actin. **(D-E)** *Left:* Immunofluorescent staining of mGluR5 in cortex **(D)** and hippocampus **(E)** regions of 263 K-infected hamsters. The images of DAPI (blue), mGluR5 (green) and merge are indicated above. *Right:* The IOD analysis for the signals of mGluR5 in the images of cortex and hippocampus sections. The IOD value of mGluR5 in control is set to 1 and *Y*-axis represents the fold changes in Ha-263 K versus control brains.

To evaluate the changes of mGluR5 in the brain tissues during prion infection, 10% brain homogenates of three scrapie strain 263 K-infected hamsters at terminal stage and three age-matched controls were evaluated by Western blots with the mGluR5-specific antibody. The blotting pattern of mGluR5 in the brain tissues was different from that in SMB cells, that beside of 130 kDa band representing the monomer, there were signals with obviously larger molecular weight mobilizing at approximately 260 kDa (Figure 7A), probably representing the dimers. Surprisingly, compared with that of healthy controls, the brain mGluR5 levels in forms of both dimer and monomer were markedly weaker in three 263 K-infected hamsters, showing significant reduction (*p* < 0.01, Figure 7A). Remarkable decreases of brain mGluR5 were also detected in the Western blots of other two scrapie-infected mouse models, 139 A- and ME7-infected mice, at end stage (Figure 7B). In addition, brain mGluR1 levels were also decreased in 263 K-infected hamsters compared with that of uninfected ones, with statistical difference (*p* < 0.05, S-Figure 2A) and mainly observed in the regions of hippocampus, cerebellum, and cortex (S-Figure 2B).

To access the alteration trend of the brain mGluR5 during prion infection, the brain specimens of 263 K-infected hamsters collected at different time points after inoculation were comparatively subjected into mGluR5-specific Western blots. Prior to the test, the brain mGluR5 levels of the young (roughly 3 weeks after weaning) and adult (12 weeks old) healthy hamsters were evaluated by mGluR5-specific Western blots, revealing quite comparable levels (S-Figure 3). Compared to that in uninoculated hamster, the signals of mGluR5 were weaker in the samples of 20 and 40 days post-inoculation (dpi) and notably weaker in that of 60 and 80 dpi (Figure 7C), highlighting that the decrease of brain mGluR5 occurs at the early stage of prion infection.

The brain sections of 263 K-infected hamsters at terminal stage were subjected into mGluR5-specific IFAs. Confocal microscopy revealed much smaller number of the mGluR5 signals (green) in the cortex (Figure 7D) and hippocampus (Figure 7E) regions of 263 K-infected hamsters than that of the controls. Further quantitative analysis indicated significant differences in the IOD value of mGluR5 per image compared with those of the controls. These results indicated that the levels of both mGluR5 and mGluR1 were decreased in the brain of scrapie-infected rodents.

### Relatively Weak Alterations of GPCR and Downstream Kinases in the Brain Tissues of 263 K-Infected Hamsters

**FIGURE 8:**
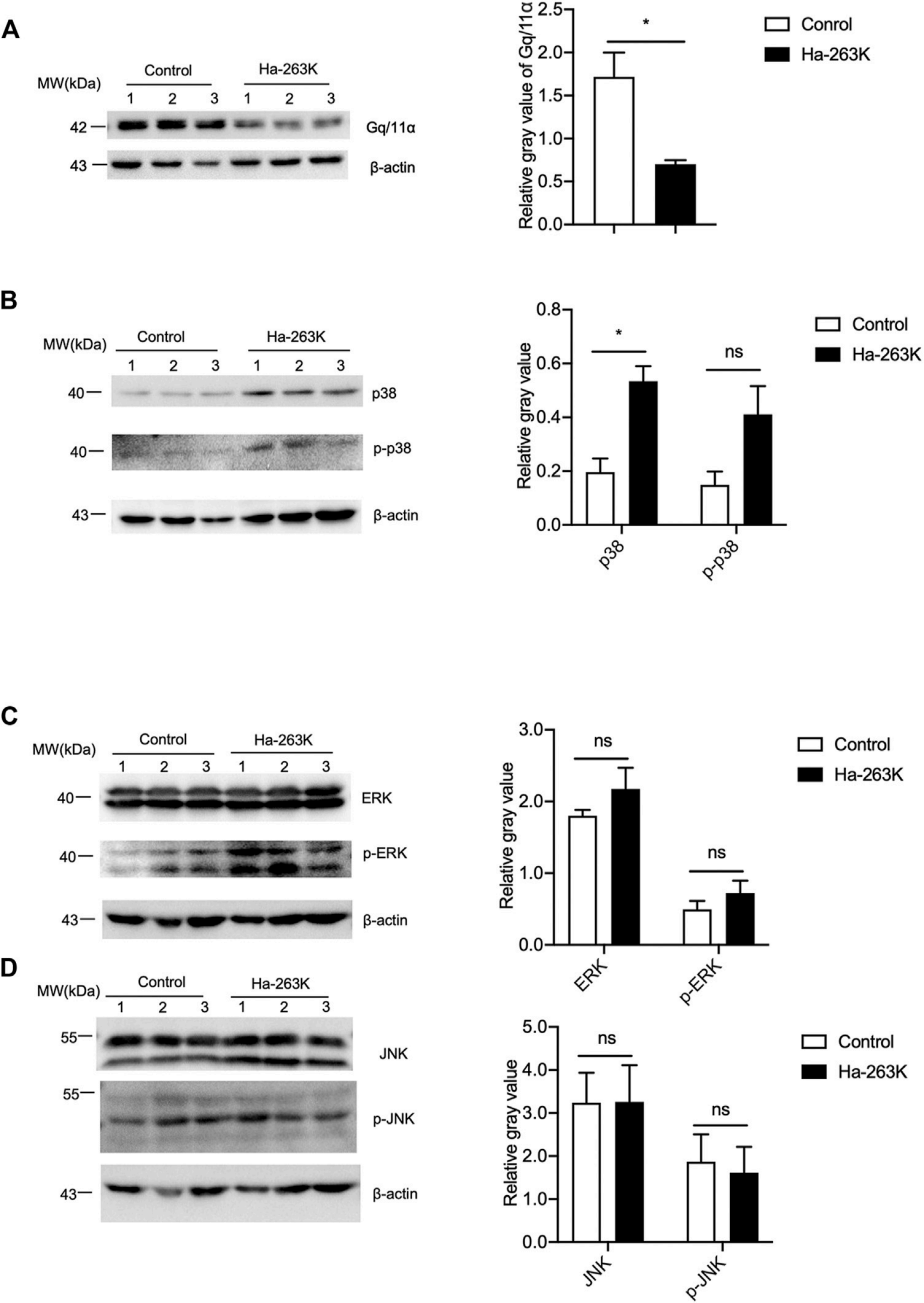
The levels of GPCR and downstream kinases in the brain tissues of 263K-infected hamsters. **(A-D)** *Left:* Western blots for the levels of Gq/11α **(A)**, p38 and p-p38 **(B)**, ERK and p-ERK **(C)**, JNK and p-JNK **(D)**. Relative molecular weights are marked on the left (1, 2, 3) indicate the number of replicates. *Right:* Densitometric analyses of the average gray values of the signals of the target proteins after being equilibrated with that of β-actin.

To see the changes in the downstream components of mGluR5 in the brains of 263 K-infected hamsters at terminal stage, the levels of Gq/11α, p38, ERK, and JNK were analyzed by individual Western blots. Compared with healthy control, the signals of brain Gq/11α in 263 K-infected hamsters were significantly weaker (Figure 8A). Both brain p38 and phosphorylated p38 in 263 K-infected hamsters were stronger than that in the controls (Figure 8B). Brain levels of ERK and phosphorylated ERK in 263 K-infected hamsters were increased compared with that of control, without statistical difference (Figure 8C). The levels of both JNK and phosphorylated JNK were comparable between healthy and infected hamsters (Figure 8D).

### Correlation of Reduction of the Brain mGluR5 With Neuron Loss in 263 K-Infected Hamsters

**FIGURE 9:**
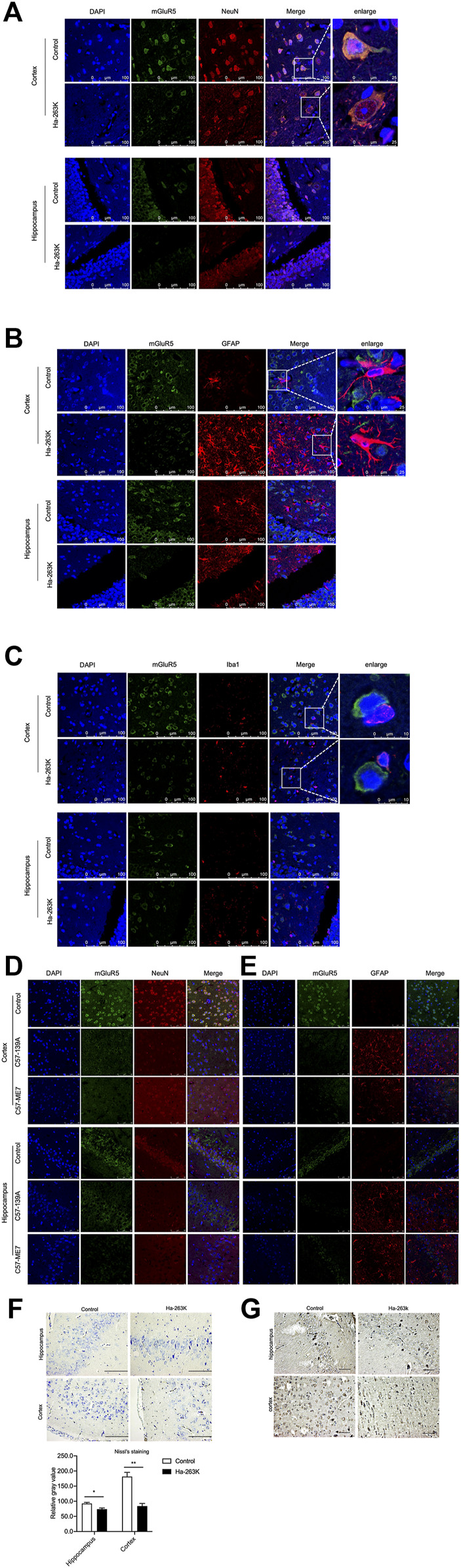
Immunofluorescent staining of mGluR5 in cortex and hippocampus slices of prion-infected rodent models. **(A-C)** Double-staining of mGluR5 with NeuN **(A)**, GFAP **(B)** and Iba1 **(C)** in cortex and hippocampus slices of 263 K-infected hamsters. **(D-E)** Double-staining of mGluR5 with NeuN **(D)** and GFAP **(E)** in cortex and hippocampus slices of 139 A-infected mice. The images of DAPI, NeuN, GFAP or Iba1, mGluR5 and merge are indicated on the top. The magnification views are shown on the right each picture. **(F)** *Top:* Nissl staining for neurodegeneration in the cortex and hippocampus regions of 263 K-infected hamsters. *Scale bar* 20 µm. *Bottom:* Quantity of neurons in cortex and hippocampus cells. **(G)** IHC assays of cortex and hippocampus regions of 263 K-infected hamsters, *Scale bar* 20 µm.

To see the localization of mGluR5 among the different types of cells in brain tissues, the brain sections from 263 K-infected hamsters at end stage and healthy controls were double-stained immunofluorescently with mGluR5 antibody, together with NeuN-, GFAP- or Iba1-specific antibodies, respectively. Among them, NeuN is a neuronal specific nuclear protein in vertebrates. GFAP is the main constituent of intermediate filaments in astrocytes and serves as a cell-specific marker that distinguishes differentiated astrocytes from other glial cells during the development in CNS. Ion calcium-binding bridle molecule 1 (Iba1) is a marker of microglia/macrophages. As illustrated in Figure 9, much less mGluR5- and NeuN-specific signals, while much more GFAP- and Iba1-specific signals were observed in the brains of the 263 K-infected hamsters, both of cortex and hippocampus. Merged pictures showed the clearly overlapped images of mGluR5 with NeuN signals (Figure 9A) in the brain sections of normal and infected hamsters, but extremely less with GFAP (Figure 9B) or Iba1 (Figure 9C) signals. Similar distributive pattern of mGluR5 was also noticed in the brain sections of normal and scrapie agents 139 A- and ME7-infected mice, that mGluR5 signals were mainly overlapped with NeuN positive cells (Figure 9D), but not with GFAP cells (Figure 9E). It seems that the mGluR5 is highly distributed in neurons, but not in the activated astrocytes and microglia during prion infection.

**FIGURE 10:**
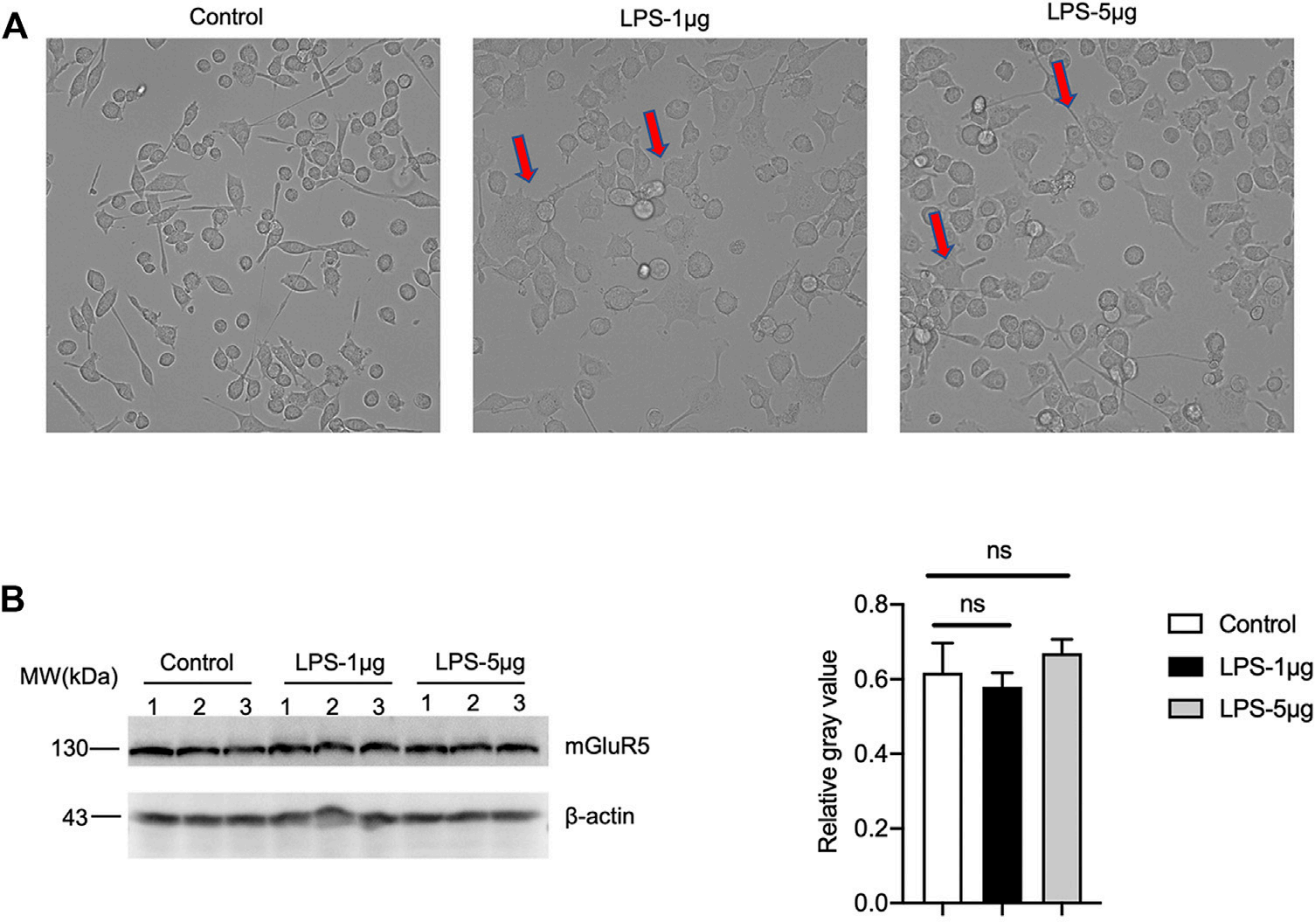
The levels of mGluR5 in LPS-stimulated BV2 cells. **(A)** The morphology of BV2 cells after LPS stimulation. **(B)** *Left:* Western blot for the levels of mGluR5 in various LPS-stimulated BV2 cells. Relative molecular weights are marked on the left. (1, 2, 3) indicate the number of replicates. *Right:* Densitometric analyses of the average gray values of the signals of the mGluR5 after being equilibrated with that of β-actin.

Neuron loss is one of the key features in prion diseases. To evaluate a possible relationship between brain mGluR5 levels and neuron numbers, the brain sections of healthy and 263 K-infected hamsters were subjected into Nissl staining and mGluR5-specific IHC, respectively. Compared to the healthy control, there were markedly less Nissl positively stained cells (blue) in the regions of cortex and hippocampus of 263 K-infected hamsters, showing statistical differences in the numbers of Nissl-stained cells per field (Figure 9F). Coincidentally, there were significantly less mGluR5 positive cells in the same brain regions of 263 K-infected hamsters (Figure 9G). In addition, the mGluR1 signals were also overlapped with NeuN positive cells in the regions of hippocampus, cerebellum, and cortex of 263 K-infected hamsters (S-Figure 4),

Since microglia also express mGluR5, to evaluate the possible difference of microglia between inactivate and activated states, a microglia cell line BV2 was stimulated with different concentrations of LPS. More round and relatively small cells were observed in the cells for 24 h after received LPS (Figure 10A). mGluR5-specific Western blots did not reveal significant difference in the mGluR5 levels in BV2 cells before and after LPS stimulation (Figure 10B). It implies that the mGluR5 levels in the cultured microglia do not change after activation. These data might highlight a linkage between decreased mGluR5 levels and neuron loss in 263 K-infected hamsters.

## Discussion

In this study, we have found for the first time aberrantly elevated levels of mGluR5 in prion-infected cell line SMB-S15, which is closely associated to the accumulation of PrP^Sc^ in the cells. Coincidentally, some elements in the downstream pathways of mGluR5, such as (GPCRs)-IP3-IP3R-Ca^2+^ and MAPK signaling pathways, were also upregulated. Notably, the increased mGluR5 and MAPK signaling levels partially recovered when removal of the prions in SMB-S15 cells by resveratrol. In contrast, we have also found that the levels of mGluR1/5 and Gq/11α are significantly reduced in the brain tissues of prion-infected rodents at the terminus of infection. Meanwhile, several kinases in MAPK signaling pathways maintain almost unchanged. Additionally, we assume that the reduced levels of mGluR1/5 in brain tissue are likely associated with the neuron loss during prion infection.

The implications of mGluR5 in the pathogenesis of many neurological diseases have been documented recently, including AD, PD, HD, autism spectrum disorders (ASD) (Zantomio et al., 2015), etc. However, there is a few of studies on the characteristics of mGluR5 in prion disease. mGluR5 interacts with a variety of proteins to participate in the regulation of neural excitatory networks, the generation of neurogenesis, and the formation of synaptic plasticity related to learning and memory (Bhattacharyya, 2016). In this study, we have proposed morphological and molecular evidence of the interaction between PrP and mGluR5 in both prion-infected SMB-S15 cells and its normal partner SMB-PS cells, as well as in 293T cells expressing abnormal Cyto-PrP. mGluR5 have been determined to interact with PrP *in vivo* and may work as a co-receptor for the complexes of Aβ oligomer and PrP^C^, and α-synuclein and PrP^C^, involving or at least partially involving in synaptic dysfunction and memory loss (Um et al., 2013; Haas et al., 2014; Beraldo et al., 2016; Ferreira et al., 2017; Brody and Strittmatter, 2018). mGluR5 interacts physically and directly with both wild-type and mutant htt (Ribeiro et al., 2014). Moreover, we have identified the increased mGluR5 that influenced the expression of mGluR1 in our experimental condition which is consistent with the results of other groups (Nakanishi et al., 1998; Mannaioni et al., 2001; Poisik et al., 2003; Kramer and Williams, 2015). It is reasonable to assume that the interaction of mGluR5 with PrP, especially with pathological PrP^Sc^, is likely to involve in the pathophysiology of prion.

On the level of a prion-infected cell model, we have noticed the overexpression of mGluR5 and IP3R, as well as the increase of intracellular Ca^2+^ in this study. Transient expression of abnormal Cyto-PrP also induces the upregulation of cellular mGluR5. Moreover, the overexpression of mGluR5 can be reverted by the removal of the propagation of cellular prions after treatment of resveratrol, while the mGluR5 level does not change in the normal partner cells in the same experimental condition, highlighting a close association of the increased cellular mGluR5 with continuous replication of prions and accumulation of abnormal PrP^Sc^. Exposure to Aβ leads to the overexpression of mGluR5 and its downstream IP3R in hippocampal astrocytes (Grolla et al., 2013). The toxic prion-mimetic compounds can increase mGluR5 clustering and accumulation at dendritic heads (Goniotaki et al., 2017). Mutant htt can sensitize IP3-mediated release of Ca^2+^ from intracellular stores (Tang et al., 2003; Ribeiro et al., 2014). It displays a scenario that accumulations of those misfolded peptides induce mGluR5 overexpression and intracellular Ca^2+^ increase. The increased mGluR5 mediated by prion propagation is convertible by the removal of prions *in vitro*.

**FIGURE 11:**
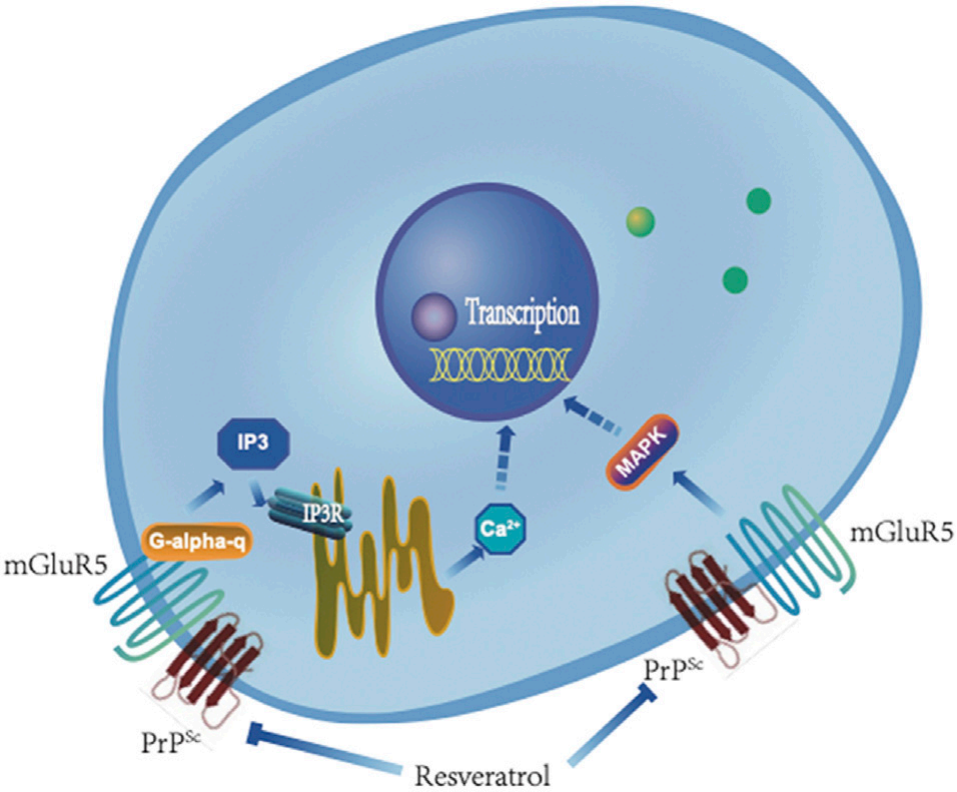
A hypothetical processing schema of GPCR-IP3-IP3R-Ca^2+^ and mGluR5-MAPK signaling pathway before and after removal of PrP^Sc^ by resveratrol.

It is well known that activation of mGluR5 mediates many biological activities. One pathway is that mGluR5 couples with Gq/11α, activates PLC, subsequently cleaves DAG and IP3, and eventually releases Ca^2+^ from intracellular stores (Figure 11). The other is that mGluR5 mediates the activations of a number of factors in MAPK pathway (Figure 11). The elevations of the downstream signaling pathways Gq/11α-IP3-IP3R-Ca^2+^ and MAPK in prion-infected cell line in this study reflect an increase of mGluR5 not only in the expression level but also in the activity during prion replication. Meanwhile, selective mGluR5 antagonist (METP) used in the present study also verifies the activation of mGluR5 increased in prion-infected cells. The abnormalities of intracellular Ca^2+^ homeostasis and the kinases in MAPK pathways have been repeatedly described in many neurodegenerative diseases including prion disease via the dysfunctions of various biological pathways (Brini et al., 2014; Ahmed et al., 2020). It is worth noting that the removal of prion propagation efficiently reduces the levels of mGluR5 and Gq/11α, but less affects the levels of phosphorylated p38, ERK, and JNK. It highlights a much closer association of increased mGluR5 with Ca^2+^ dysregulation.

The distribution of mGluR5 in CNS tissues is considered wide both in neuronal and non-neuronal cells, including astrocytes, microglia, oligodendrocytes, and stem cells (Balazs et al., 1997; Biber et al., 1999; Kumar et al., 2015). However, our IFA assays of brain sections have illustrated that mGluR5 signals, particularly the increased mGluR5 signals in the scrapie-infected experimental hamsters and mice, mainly colocalize with neurons. Furthermore, mGluR1, the other subtype of mGluR group I, also colocalize with neurons. Meanwhile, our data here have proposed that over activation of mGluR5 contributes to the cell damage *in vitro*. It may let us speculate that the overexpression of mGluR5 at early stage of prion infection influences the survival of cells. With the progression of the disease, a large number of neurons were lost, resulting the levels of mGluR1/5 were significantly reduced at the late stage of disease. However, it cannot be ruled out that other factors are involved in nerve damage, especially in the early stages of prion infection. Although mGluR5 is detectable in cultured BV2 cells, stimulation of BV2 cells with LPS seems not to affect the mGluR5 level. Astrocytic mGluR5 is believed to involve in glia-neuron interactions, regulation of glutamate reuptake, and the coupling of the neurovascular to neuronal activity (Vermeiren et al., 2006; D'Ascenzo et al., 2007). Increased mGluR5 in astrocytes is reported in several acute and chronic neurodegenerative conditions, e.g., epilepsy, brain injury, amyotrophic lateral sclerosis, and multiple sclerosis, which contribute selectively to the apoptosis of astrocytes (Paquet et al., 2013). A more detailed study has demonstrated different reactive phenomena of astrocytes collected from different brain regions to the exposure of Aβ, that the astrocytes from hippocampus show the increased mGluR5 and InsP3R while those from entorhinal cortex fail (Grolla et al., 2013). Contrary to above observations, we notice extremely less colocalization of mGluR5 with the proliferative astrocytes in the regions of cortex and hippocampus from three scrapie agents infected rodent models. Although we cannot exclude the limitation from the point experimental technique, the alteration of mGluR5 in the brains of prion-infected animals seems to be less associated with the proliferated astrocytes and activated microglia. Further assays with different mGluR5 antibodies may help to address those differences.

Unexpectedly, the brain mGluR5 levels of three scrapie-infected rodent models are remarkably decreased at the end stage in this study, even, the reduction of brain mGluR5 occurs at early stage of prion infection. Furthermore, decreased mGluR1 levels are also observed in brains of 263 K-infected hamsters. The brain Gq/11α in scrapie agent infected animals also decreases. Such alteration of brain mGluR5 in scrapie-infected experimental rodents seems to be contrary to the observations in prion-infected SMB-S15 cells. Our previous studies have demonstrated many proteins in SMB-S15 cells that are abnormally changed, which display similar changing trends as prion-infected individuals. However, we have also identified that lots of abnormally regulated brain proteins in prion-infected animals do not significantly change in SMB-S15 cells or change in opposite direction, such as αΒ-crystalline, brain-derived neurotrophic factor (BDNF) and the relevant factors (TrkB, p-TrkB, GRB2 and p57NTR), metalloproteinase (ADAM10), glucose transporter 3 (GLUT3), and Polo-like kinases 3 (PLK3) (Wang et al., 2013; Chen et al., 2014; Yan et al., 2014; Ting-Ting Wang et al., 2016; Wang et al., 2017). Moreover, majority of the abnormally expressed proteins in SMB-S15 cells can be completely or partially converted by removal of prion replication with resveratrol, but some do not, such as RyR2 and caspase 8 (Shi et al., 2018; Ma et al., 2019). We assume that although prion-infected cell lines mimic to some extent the prion infection *in vivo*, it more reflects a situation that the cells adopt the propagation and accumulation of prions; thus, the protein changing profile in prion-infected cell line may differ with that in the brains of prion-infected animals.

Early studies have illustrated a highly expressed mGluR5 in the brain regions of some neurodegenerative diseases, e.g., AD, PD, and HD. The expression of mGluR5 is also markedly elevated in caudate nucleus of normal elderly individuals (Tsamis et al., 2013). PET imaging assay has showed an elevated brain mGluR5 in LPS-induced murine neuroinflammation model and in the brains of AD and ALS patients (Muller Herde et al., 2019). mGluR5 signaling may lead to the activation of either neuroprotective pathways or neuronal toxicity. Recently, the roles of the interaction mGluR5 with Aβ and α-synuclein-PrP^C^ complexes in mediating the pathogenesis and progression of AD and PD attract great attention (Overk et al., 2014; Ferreira et al., 2017; Brody and Strittmatter, 2018; Lee et al., 2018). Therapeutic intervention designed to disrupt Aβ oligomer-PrP^C^ signaling through mGluR5 become a pharmacological hot point (Brody and Strittmatter, 2018). The impairment of mGluR5 signaling in CNS tissues of prion disease is poorly understood. Although the results of three scrapie-infected rodent models here show a reduced brain mGluR5 which is speculated to be associated with relatively rapid neuron loss, more studies with human and other animal prion diseases are expected to get deep understanding of the alteration of brain mGluR5 in prion disease.

## Data Availability

The original contributions presented in the study are included in the article/Supplementary Material, further inquiries can be directed to the corresponding authors.

## Supplementary Material

Supplementary Figure S1 The level of mGluR1 in SMB-PS and -S15 cells as well as CHPG treated SMB-PS cells. **(A)** *Left*: Western blot for the levels of mGluR1 in SMB-PS and SMB-S15. Relative molecular weights are marked on the left. *Right*: Densitometric analyses of the average gray values of the signals of the mGluR1 after being equilibrated with that of β-actin. **(B)** *Left*: Western blot for the levels of mGluR1 in CHPG treated SMB-PS cells. *Right*: Densitometric analyses of the average gray values of the signals of the mGluR1 after being equilibrated with that of β-actin.

Supplementary Figure S2 The level of mGluR1 in brains of 263K-infected hamster at end stage. **(A)** *Upper*: Western blot for the levels of brain mGluR1 in control and Ha-263K. Relative molecular weights are marked on the left. *Lower*: Densitometric analyses of the average gray values of the signals of the mGluR1 after being equilibrated with that of β-actin. **(B)** Immunofluorescent staining of mGluR1 in cortex, cerebellum and hippocampus regions of 263K-infected hamsters. The images of DAPI (blue), mGluR1 (green) and merge are indicated above. The IOD analysis for the signals of mGluR1 in the images of cortex, cerebellum and hippocampus sections are indicated on the right.

Supplementary Figure S3 The level of mGluR5 in brain tissues of 3 weeks and 12 weeks old healthy hamsters. *Left*: Western blot for the levels of mGluR5 in brains of two age groups. Relative molecular weights are marked on the left. *Right*: Densitometric analyses of the average gray values of the signals of the mGluR5 after being equilibrated with that of β-actin.

Supplementary Figure S4 Double-staining of mGluR1 with NeuN in cortex, cerebellum and hippocampus slices of 263K-infected hamsters at end stage. The images of DAPI, NeuN, mGluR5 and merge are indicated on the top. The magnification views are shown on the right each picture.
