# Supplementary figures and images for: Different Aberrant Changes of mGluR5 and Its Downstream Signaling Pathways in the Scrapie-Infected Cell Line and the Brains of Scrapie-Infected Experimental Rodents
Source: Front Cell Dev Biol. 2022 May 12;10:844378. doi: 10.3389/fcell.2022.844378 (PMC9133610; doi:10.3389/fcell.2022.844378)

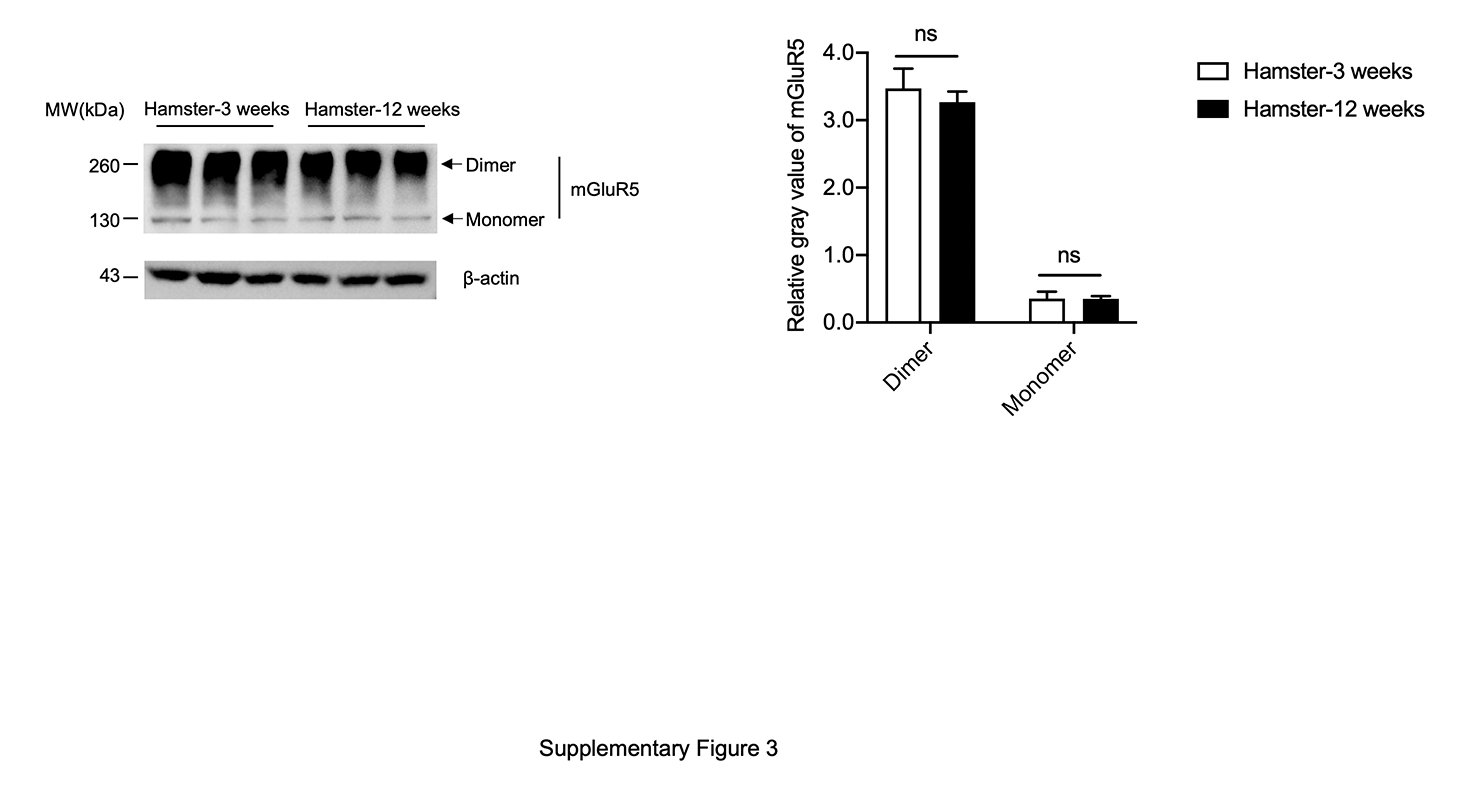

Supplement: Supplementary file 1 [file Image3.TIF]

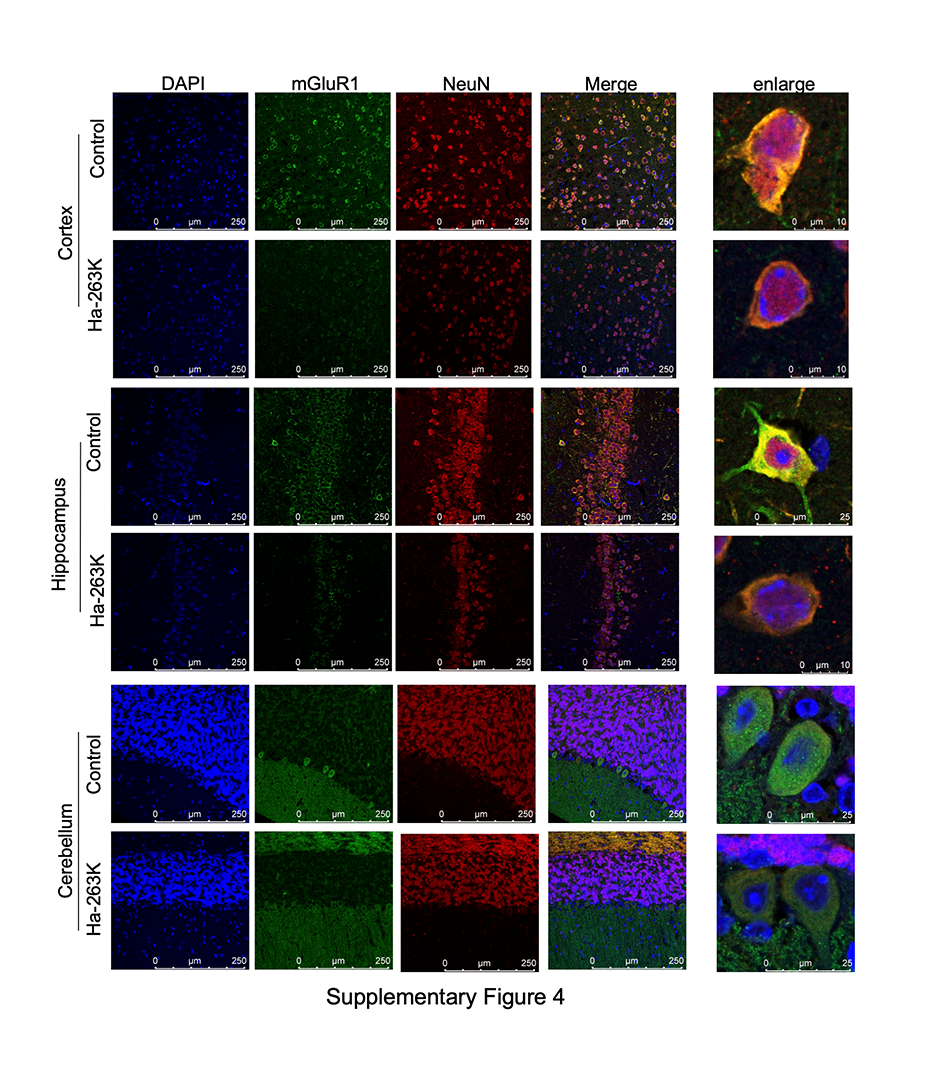

Supplement: Supplementary file 2 [file Image4.TIF]

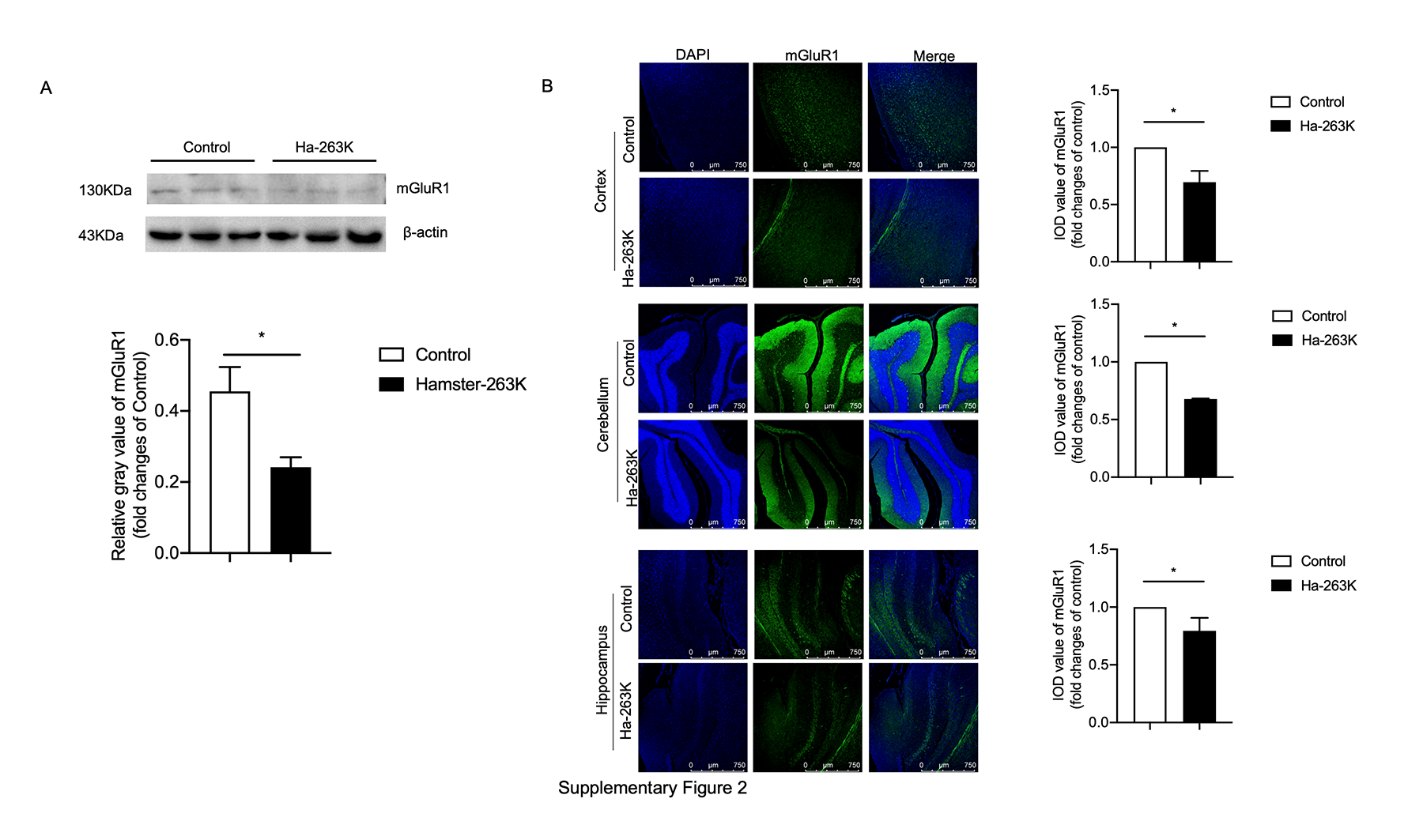

Supplement: Supplementary file 3 [file Image2.TIF]

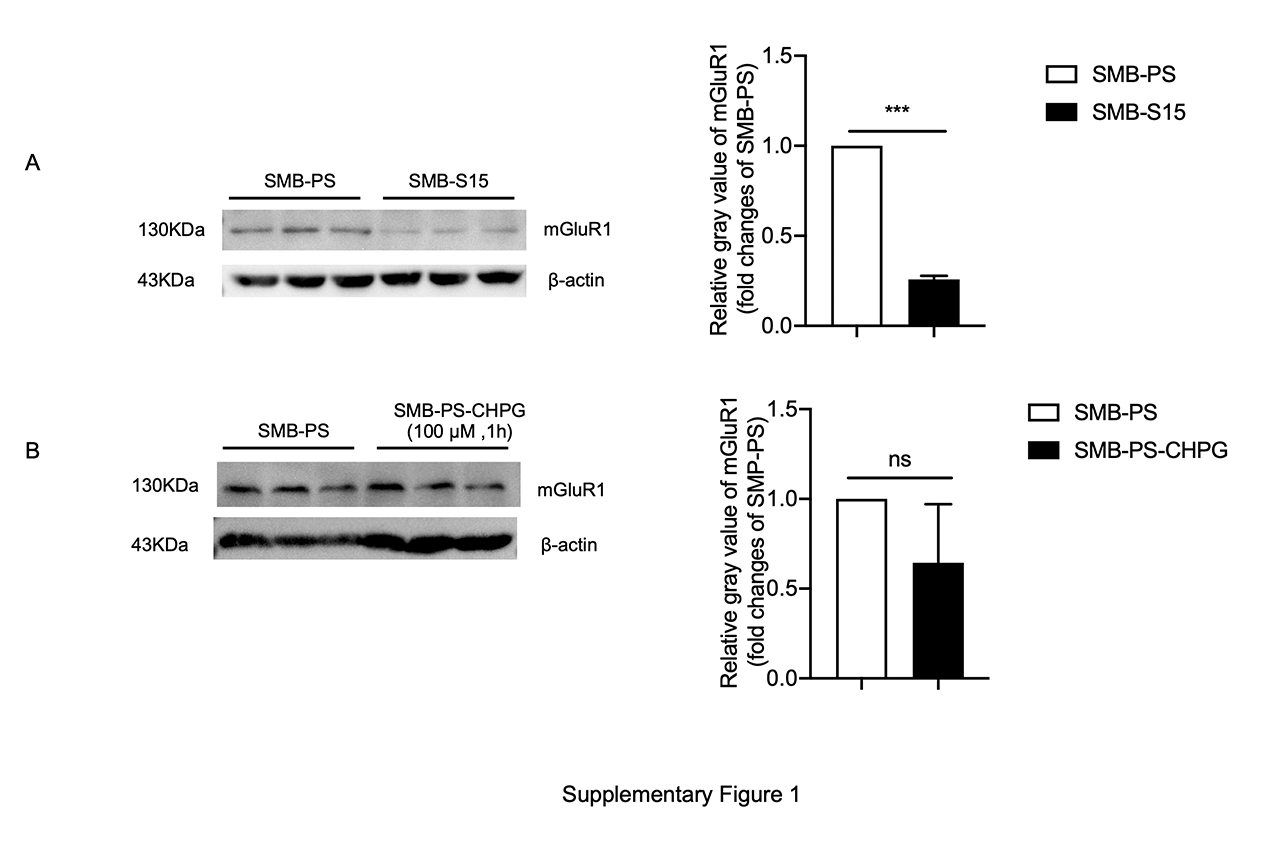

Supplement: Supplementary file 4 [file Image1.TIF]
